# Assessing the Impact of Silage Inoculants on the Quality of Adina Alfalfa Silage

**DOI:** 10.3390/microorganisms13040841

**Published:** 2025-04-07

**Authors:** Siyi Wang, Zhennan He, Heng Jiang, Le Sun, Guolin Yang, Yuanyuan Jing, Fengqin Gao

**Affiliations:** 1 Institute of Grassland Research Chinese, Academy of Agricultural Science, Hohhot 010010, China; 17860788357@163.com (S.W.); a2964898754@163.com (Z.H.); 82101215199@caas.cn (H.J.); 15066745843@163.com (L.S.); 16634230300@163.com (G.Y.); 2 College of Grassland Science, Qingdao Agricultural University, Qingdao 266109, China

**Keywords:** Adina alfalfa, silage inoculants, fermentation quality, nutritional quality, membership function analysis

## Abstract

In order to explore the effects of different silage inoculants on the silage quality of alfalfa (*Medicago sativa* L.), this study utilized six groups of experimental treatments and five kinds of additive treatments: Xinlaiwang I straw silage (group A), Xinlaiwang I alfalfa silage (group B), Zhuanglemei silage starter culture (group C), Baoshiqing (group D), Kangfuqing S lactic acid bacteria silage (group E), and another blank control group (CK group, distilled water). The effect of silage on fermentation characteristics and nutritional value of Adina alfalfa silage was studied by membership function analysis. The main study variable was inoculant strains. Alfalfa silage was packed into polyethylene plastic vacuum bags in the laboratory and sealed for 60 days. The silage was divided into six treatment groups with three replicates per group. The fermentation performance and nutrient composition of the silage were determined. The results showed that compared with the control group, adding Xinlaiwang I alfalfa silage (group B) could significantly increase the contents of crude protein (CP) and lactic acid (LA) in alfalfa silage (*p* < 0.05), decrease the contents of neutral detergent fiber (NDF) and acid detergent fiber (ADF), and decrease the pH and ammoniacal nitrogen/total nitrogen (AN/TN). The results showed that different inoculants could improve the silage quality of alfalfa to different extent, and Xinlaiwang I alfalfa silage had the best effect.

## 1. Introduction

The global alfalfa cultivation area is approximately 322 million hectares (hm^2^), of which the United States, Russia and Argentina account for approximately 70% of the planted area. In the “14th Five-Year Plan” National Forage Industry Development Plan [1] issued by the Ministry of Agriculture and Rural Affairs of China in 2022, it is clear that by 2025, China’s high-quality forage production will reach 98 million tons, and the feed demand guarantee rate of cattle and sheep will reach more than 80%. The scientific and complete use of high-quality forage resources is key to ensuring the steady supply of forage and to realizing an income increase for farmers and herdsmen [2]. Alfalfa (*Medicago sativa* L.) belongs to the leguminous crop type, which is rich in crude protein (CP) and rich in vitamins and minerals in the stems, leaves, and seeds [3]. It has high nutritional value, high grass yield, good palatability, and well-developed roots, which can absorb water and nutrients from the deep soil and help reduce surface runoff. It has the effect of protecting water and soil [4], and its characteristics of cold resistance, drought resistance, salt and alkali resistance, strong biological nitrogen fixation ability, strong stress resistance, and good regeneration abilities give it a wide range of adaptability, and it has been vigorously promoted worldwide.

Alfalfa can be used as feed not only for direct green feeding or grazing but also for silage, green hay, granule, etc., which are widely used in dairy cattle breeding. The use of alfalfa in feed can significantly enhance the production performance of animals [5] and improve the quality and production performance of animal products. In addition to being processed into silage, hay, or powdered feed, it can also be mixed with gramineous forage to achieve complementarity [6] and obtain better benefits. Some scholars have shown that adding fermented alfalfa to livestock and poultry diets can significantly improve production performance, improve animal product quality, and increase economic benefits. Silage inoculants can improve the palatability and nutritional value of feed, make animals more willing to eat, and improve the utilization rate of feed. Silage technology allows feed to be stored during periods of abundant resources for use when resources are scarce, achieving a stable feed supply throughout the year. The silage of high-quality forage is a simple and effective way to alleviate feed shortages for ruminants [7]. Lactic acid bacteria are key components of silage fermentation and play a key role in silage quality [8]. Zhang et al. [9] showed that formic acid, acetic acid, and propionic acid (PA) inoculants had positive effects on the fermentation and nutritional quality of oat silage. Adding lactic acid bacteria and other microbial preparations can inhibit the growth of various bacteria in the silage process, shorten the silage time, increase palatability [10], and improve the quality of silage. Silage has very broad application prospects in animal husbandry.

The direct effects of silage inoculants such as Xinlaiwang I straw silage, Xinlaiwang I alfalfa silage, Zhuanglemei silage starter culture, and Baoshiqing and Kangfuqing S lactic acid bacteria silage on the fermentation characteristics and nutritional value of alfalfa silage were studied by means of silage belonging function analysis. This study assumed that compared to other treatment groups, Xinlaiwang type i alfalfa silage was the most effective silage agent compared with other silage agents and aimed to identify the most suitable additives in the quality production of alfalfa silage and provide a theoretical basis for the high quality production practice of alfalfa silage and the promotion and utilization of high-quality probiotic inoculants.

## 2. Materials and Methods

### 2.1. Experiment Site Profile

The experiment was conducted at the alfalfa planting base of Knights Dairy in Erdos, Inner Mongolia (Dalat Banner, Inner Mongolia Autonomous Region), on 10 July 2020, and three crops of alfalfa were planted (*Medicago sativa* cv. Adina). The three crops of alfalfa planted at 110°02′25″ E and 40°28′48″ N were used as the base material. The test area was located in a typical temperate continental monsoon climate, with an average annual temperature of 6.8 °C, an extreme minimum temperature of –309 °C, a frost-free period of 135 days, and an average annual precipitation of 320 mm. The growing season (April–September) accounted for 89% of the annual precipitation. The altitude was 1010 m, the soil was chestnut soil, the content of organic matter was 4.86 g/kg, the available potassium was 94.65 mg/kg, the available phosphorus was 10.46 mg/kg, the alkali–hydrolytic nitrogen level was 11.15 mg/kg, and the pH value was 8.2. Sprinkler irrigation conditions were used to negate drought and keep the soil low in salt and other pollutants. The relative humidity of the air was 87%, the precipitation was 0 mm, and mowing was carried out at the beginning of the flowering stage, when the stubble height is usually between 7 and 10 cm. The drying process took place naturally, with the cut plants spread out in a well-ventilated, sunny area. They were turned regularly to accelerate water evaporation and ensure even drying. Drying time depended on weather conditions and the water content of the plant; drying was carried out until the water content was about 60%. At this point, the sensory performance of the water content should be tested as follows: squeeze a plant with your hand, and if no water flows out out, the water content is just right and is conducive to the fermentation of bacteria and increasing the palatability of the feed. During the experiment, the weather conditions were mainly sunny with occasional showers, which was conducive to the growth of crops and to the experiment.

### 2.2. Raw Materials and Additives

The nutritional components of raw alfalfa, as shown in Table 1.

The formula for calculating the *RFV* usually involves the dry matter (*DM*), crude protein (*CP*), acid detergent fiber (*ADF*), and neutral detergent fiber (*NDF*) in the feed. The specific calculation method is defined as follows:

*RFV* = (*DDM* × *DCP*)/(*ADF* + *NDF*)

Here, *DDM* is the digestible dry matter content in dry matter, *DCP* is the crude protein content in dry matter, *ADF* is the acid detergent fiber content, and *NDF* is the neutral detergent fiber content.

### 2.3. Silage Production

Six treatments and five inoculants were used in the experiment. The sources, compositions, and contents of silage inoculants are shown in Table 2. According to their mass ratios, Xinlaiwang I straw silage (A), Xinlaiwang I alfalfa silage (B), Zhuanglemei silage starter culture (C), and Baoshiqing (D) and Kangfuqing S lactic acid bacteria silage (E) were added, respectively, and distilled water was used as the control treatment (CK group). The cut alfalfa was dried to about 60% water content, then was cut short to 1–2 cm, and the inoculants were mixed together evenly and completely and were placed in vacuum silage bags (200 mm × 250 mm). Each bag was filled with about 300 g, was vacuum-sealed with a vacuum machine, and was stored at room temperature. The experiment utilized six treatments with three repetitions for each treatment. After 60 days of silage fermentation, 20 g of silage were removed per bag.

### 2.4. Fermentation and Nutritional Quality Determination

After fermenting the silage for 60 days, the mold-deteriorated silage was removed. The remaining silage was sampled at multiple points, and 20 g of silage was taken from each bag, after being fully mixed. A total of 180 mL distilled water was added, the silage was mixed evenly, ground for 1 min using a juicer, and filtered through four layers of gauze and qualitative filter paper. It was then left to stand at 4 °C in a refrigerator for 24 h. The fermentation index was measured by the extract. Lactic acid is the main organic acid produced by the fermentation of sugars in silage through lactic acid bacteria. Its production helps to reduce pH value, inhibit the growth of harmful microorganisms, and maintain the nutritional value and stability of feed. Acetic acid is produced during silage by *Acetobacteria*, *Bacillus*, *Escherichia coli*, and certain *Klebsiella*, *Lactobacillus*, and *Pediococcus* species and helps to prevent feed rot and mold growth. PA has the effect of inhibiting the growth of mold and yeast, especially in the storage process of silage, helping to maintain a dry and stable feed. Butyric acid is a four-carbon carboxylic acid produced by certain anaerobic bacteria during fermentation. It is often associated with poor silage conditions. Its high content may indicate the poor fermentation of feed, resulting in decreased nutritional value and the poor palatability of feed. AN (ammoniacal nitrogen) refers to nitrogen in the form of ammonia, which is the product of protein degradation. AN content is an important index for evaluating the quality of silage. A high AN content may indicate the excessive protein degradation and reduced nutritional value of feed. TN (total nitrogen) content refers to the sum of all forms of nitrogen in the sample, including organic nitrogen, ammonia nitrogen, nitrate nitrogen, etc. TN content is an important parameter for measuring the nutritional value of silage, reflecting the total content of protein and amino acids in the feed.

The determination of the contents of lactic acid (LA), acetic acid (AA), PA, and butyric acid (BA) is performed using high-performance liquid chromatography (HPLC), and the specific operation is carried out in a high-performance liquid chromatograph device: the samples are acidified, distilled to from the extract, centrifuged or filtered to remove large molecules, and then derivatized and analyzed (refer to the method in standard DB15/T1458-2018 [11] for details). The ratio of AN (AN) to TN (TN) content is calculated as follows: TN content = crude protein/6.25. A V-score system was used to evaluate the fermentation quality of alfalfa silage [12].

The nutritional quality of the 100 g samples was determined after baking in an oven at 65 °C for 48 h and then crushing with a grinder. Dry matter (DM) was determined by drying method, referring to the standard GB/T6435-2014 [13]. Crude protein (CP) content was determined using reference standard GB/T6432-2018 [14]. Crude ash (Ash) content was determined using reference standard GB/T6438-2007 [15]. Neutral detergent fiber (NDF) and acid detergent fiber (ADF) were determined using reference standard GB/T20806-2006 [16]. Water soluble carbohydrate (WSC) content was determined using the method of Xu Meirong et al. [17], and filtrate pH was determined using a LAQUAtwin-pH-22 handheld precision pH meter [18].

### 2.5. Data Statistics and Analysis

Excel 2021 and SPSS 26.0 were used for table drawing and one-way ANOVA, and Duncan’s method was used for multiple comparisons. The results were expressed as “means ± standard error”, and *p* < 0.05 indicated significant differences.

Membership function method was used to measure the membership function value of each index of the silage; this was then was summarized, and the silage quality of various inoculants was comprehensively ranked through calculations. The value of silage membership function ($U(X_{j})$) is the scale used to evaluate the quality of silage. The higher the value, the better the quality of the silage. Among the many evaluation indexes, the contents of DM, CP, crude fat, starch and lactic acid, as well as the degradation rate of neutral detergent fiber in 30 h, were significantly positively correlated with the quality of silage. Therefore, the formula for calculating the value of the subordinate function of these substances is as follows:

$$U(X_{j})=\frac{X_{j}-X_{min}}{X_{max}-X_{min}}$$

The contents of pH, neutral detergent fiber, acid detergent fiber, ammonia nitrogen, and acetic acid are negatively correlated with silage quality. The membership function value is calculated by the following formula:

$$U(X_{j})=1-\frac{X_{j}-X_{min}}{X_{max}-X_{min}}$$

In the formula, *U(*$X_{j}$*)* represents the membership function value of a measurement index; $X_{j}$ represents the measured value of this indicator; $X_{max}$ indicates the maximum value of this indicator; and $X_{min}$ indicates the minimum value of this indicator.

Membership function involves a fuzzy linear regression model. Fuzzy linear regression models are a type of fuzzy regression model which are based on the linear representation of structural elements, with an input of accurate data and an output of fuzzy data. The model determines the regression coefficient and ambiguity function in the least squares and is suitable for dealing with the fitting degree between the observed value and the predicted value.

## 3. Results and Analysis

### 3.1. Effects of Different Silage Inoculants on the Nutritional Quality of Alfalfa Silage

As can be seen from Table 3, compared with the control group, crude protein contents in groups A, B, D, and E were significantly higher than in the control group (*p* < 0.05), The neutral detergent fiber content of group B was significantly lower than that of other groups (*p* < 0.05), and the ADF content of group B was lower than that of other groups, but there was no significant difference (*p* > 0.05). In addition, the contents of DM and the relative feed value (RFV) in group B were slightly increased compared with the other five groups, but there were no significant differences (*p* > 0.05). Water soluble carbohydrate in group B also increased compared with the other five groups, and there were significant differences between group A and group D (*p* < 0.05).

### 3.2. Effect of Inoculants on Fermentation Quality of Alfalfa Silage

It can be seen from Table 4 that the inoculants significantly affected the contents of pH, AA, and LA in alfalfa silage (*p* < 0.05). The pH of silage treated with different inoculants was higher than that of group B. The AN content in group D was significantly higher than that in groups E and B (*p* < 0.05). The content of LA in group B was significantly higher than that in other groups (*p* > 0.05). In general, diets with better silage quality should have a lactic acid content of between 3.0% and 13% (DM), a butyric acid content of <0.2% (DM), and an ammonia nitrogen/TN content of less than 10%. According to this standard, in this experiment, the lactic acid content of the B, D, and E treatment groups at 60 days of silage was greater than 3.5%, the histamine nitrogen/TN content in all treatments was greater than 10%, and PA and butyric acid were not detected, which met the requirements of high-quality silage. The ratio of lactic acid to acetic acid is usually used as an indicator to evaluate the effectiveness of lactic acid fermentation, and it is generally believed that the ratio should not be lower than 3:1, and the higher the ratio, the better the silage quality. When the ratio of lactic acid to acetic acid is lower than 3:1, this indicates that the number of homofermented lactic acid bacteria is low and the number of lactic acid bacteria is insufficient. In this experiment, the ratio of lactic acid to acetic acid in all treatment groups was less than 3:1, indicating that the number of lactic acid bacteria could not guarantee the fermentation of silage, and the fermentation mode was the homotype fermentation mode.

Lactic acid is the main organic acid produced by the lactic acid bacteria fermentation of sugars in silage. Its production helps to reduce pH value, inhibit the growth of harmful microorganisms, and maintain the nutritional value and stability of the feed.

Acetic acid is produced during silage by *acetobacteria*, *Bacillus*, *Escherichia coli*, and certain *Klebsiella*, *Lactobacillus*, and *Pediococcus* species and helps to prevent feed rot and mold growth. PA has the effect of inhibiting the growth of mold and yeast, especially in the storage process of silage, helping to maintain dry and stable feed.

Butyric acid is a four-carbon carboxylic acid produced by certain anaerobic bacteria during fermentation. It is often associated with poor silage conditions. Its high content may indicate the poor fermentation of feed, resulting in decreased nutritional value and the poor palatability of feed.

AN refers to nitrogen in the form of ammonia, which is the product of protein degradation. AN content is an important index for evaluating the quality of silage. High AN contents may indicate excessive protein degradation and the reduced nutritional value of feed.

TN content refers to the sum of all forms of nitrogen in a sample, including organic nitrogen, ammonia nitrogen, nitrate nitrogen, etc. TN content is an important parameter for measuring the nutritional value of silage, reflecting the total contents of protein and amino acids in the feed.

### 3.3. Comprehensive Analysis on the Quality of Alfalfa Silage Treated with Different Lactic Acid Bacteria

The membership function analysis of 10 core indexes of alfalfa silage was carried out, and the results are shown in Table 5. The larger the average membership function value, the better the comprehensive performance and the better the silage quality. The average membership function values were sorted, and the results show that group B has the largest membership function value with an average membership function value of 0.69, followed by group E, with an average membership function value of 0.61, and group D, with an average membership function value of 0.58. Group C had a limited effect on alfalfa silage with an average membership function value of 0.24. Membership function analysis is a simple, accurate, and fast comprehensive evaluation method that uses fuzzy mathematics principles, evaluates and studies various indexes, and assesses material properties comprehensively. Today, this method is being increasingly frequently applied to forage variety screening and quality evaluation.

## 4. Discussion

### 4.1. Effect of Inoculants on Nutrient Composition of Alfalfa

The concentration of DM during silage is one of the key indexes used to evaluate the nutritional value of silage. During the entire process of silage fermentation, variations in DM concentration are mainly due to the respiration activities of plants and the activity of microorganisms in certain aerobic environments [19,20,21]. In the silage of each treatment group in this study, compared with the CK group, the DM content of each probiotic addition group was increased, which was consistent with the results of previous studies [22], indicating that the inoculation of probiotic additions can effectively prevent the degradation of DM by microorganisms during silage.

The Alfalfa used in this study has a significantly higher CP content and is used as a high-quality protein feed resource, but its low WSC content and high buffer energy make it a poor choice for sole use in the silage fermentation process. Therefore, inoculants are considered to ensure its fermentation quality. Structural carbohydrate affects the fermentation quality, digestibility, and palatability of feed. However, some inoculants can be broken down into dissolved sugars that are easy to use, such as cellulose and hemicellulose, in order to reduce the structural carbohydrate content of silage and improve fermentation quality and other physicochemical factors [23]. In this experiment, group B inoculants exhibited better silage quality, which may be related to more structural carbohydrates decomposed by microorganisms in the inoculants. In the present study, the addition of Xinlaiwan I alfalfa silage had no significant effect on the NDF and ADF contents of alfalfa silage but only reduced the NDF and ADF contents to a certain extent, which may be because most commercial lactic acid bacteria lack the activity of the hydrolyzing plant cell wall polynanase. Our experimental results were different from the conclusions of Liu et al. [24]., where the addition of lactobacillus had no significant affect on the rumen disappearance rates of NDF and ADF in silage compared with the control group, which may be related to different material compositions and experimental designs.

As a substrate in the fermentation process of silage, the content of WSCs determines the degree of silage fermentation. In this experiment, the WSC contents in the probiotic addition groups A, C, and D were lower than in the CK group, which might be because the inoculations accelerated the utilization of glucose and other WSCs. The WSC contents of groups B and E were higher than in other groups, and the DM content was increased, which was consistent with the results of Zi et al. [25]. This might be because the probiotics in these two groups of inoculants were able promote the degradation of structural carbohydrates, thus increasing the WSC content. Although the WSC contents of groups B and E were increased compared with that of the CK group, there were no significant differences, and it was observed that the WSC contents of groups B and E with low fiber contents were higher. Compared with the control group, the inoculants in group B were able to significantly reduce the NDF content (*p* < 0.05) and effectively reduce the ADF content, which was similar to the results seen in the study by Eri ADCI [26]. It can be inferred that the reason for this result may be due to the treatment of certain probiotics in the group B inoculants and the fact that the pH of the silage is relatively low, which enables it to undergo the stronger acid hydrolysis of fibers. Not only that, but probiotics can also secrete ferulate esterase, α-amylase, etc.; these enzymes and bacteriocin can decompose the ester bonds between hemicellulose and lignin [27]. The degradation of NDF and ADF was enhanced. This may be due to the fact that the subtilis and *L. brucei* in the inoculants supplement *L. brucei’*s consumption of sugar sources by promoting the degradation of structural carbohydrates and increasing the content of WSCs [28]. In order to further explore how to optimize the overall quality of alfalfa silage, Yan et al. [29] showed that when the raw material water content was 55%, alfalfa treated with 0.4% PA had the best comprehensive performance and was able to effectively improve the quality of alfalfa silage.

The results of this study showed that the nutritional components of group E and the control group were similar and that there were no significant differences in NDF, ADF, WSC, and RFV levels. In addition, Yang et al. [30] found that high water content in wilting alfalfa silage led to the rapid consumption of water-soluble carbohydrates (WSCs) and the growth of harmful microorganisms, resulting in poor fermentation quality. Wilting treatment and the inoculation of alfalfa silage could improve the fermentation quality of alfalfa silage and inhibit the growth of spoilage microorganisms and achieve the best fermentation quality of silage. In this experiment, the WSC content in the control group was higher than that in group B, which may be due to the higher water content. 

This study showed that compared with the control group, although the content of ADF decreased and the contents of DM and CP increased in each additive treatment group, there were no significant differences. The result of this experiment is very similar to the conclusions of previous research data, which may be due to the different effects of different inoculants during the processing of silage of various raw materials.

### 4.2. Effects of Inoculants on Fermentation Characteristics of Alfalfa Silage

This experiment showed that the inoculants in group B significantly reduced the pH and AN/TN of silage, increased the lactic acid content significantly, and reduced the dry matter loss of the silage significantly, which was consistent with the results of previous studies. After adding lactic acid bacteria and cellulase, the pH of the silage decreased and the lactic acid content increased, which effectively improved the fermentation quality and revealed good storage characteristics. Muck et al. [31] found that the addition of *Lactobacillus Plantarum (LP)* and *Lactobacillus Brucei* (*LB*) were beneficial for alfalfa silage, as these reduced pH and protein degradation and inhibited the proliferation of harmful microorganisms, which is similar to the results of this experiment. It can be speculated that the results of this experiment may have been caused by the presence of such microbial flora in group B inoculants. Moreover, Aydin et al. [32] showed that the addition of PFJ (3% sucrose) prepared with 2% honey clover to alfalfa silage had positive effects on alfalfa silage, fermentation characteristics, and in vitro organic matter digestibility (IVOMD). In addition, Elferink et al. [33] found that *Lactobacillus brucei* could decompose lactic acid into acetic acid, PA, and 1, 2-propylene glycol in an anaerobic environment. dos Santos et al. [34] showed that the use of lyophilized or activated *Lactobacillus brucei* in silage could significantly increase the number of lactic acid bacteria in corn silage throughout the entire fermentation process. The study by Li et al. [35] showed that the inoculation of *Lactiplantibacillus plantarum* A1 (Lp A1), which produces ferulic acid esterase (FAE), into silage could effectively improve its quality and digestibility, regulate rumen fermentation, and increase the utilization rate of the feed. In view of this, it can be speculated that, in the future, the fermentation quality of silage inoculants could be improved by inoculating Lp A1 and adding sucrose solution to some extent.

In fact, during the silage process, the decomposition of CP is mainly caused by the action of plant proteases and the activities of aerobic microorganisms [36], resulting in the generation of non-protein nitrogen, AN, and amine compounds [37]. However, with the decrease in pH value, the activity of plant protease and microbial activity may be inhibited, which helps to slow down the protein hydrolysis process [38]. In this study, the CP content of all probiotics in the five silages exceeded that of the CK group, while the AN/TN of group B and E was significantly lower than that of the CK group, which proved that both inoculants could effectively maintain protein to a certain extent and inhibit protein hydrolysis processes [39].

In this experiment, the pH of all groups was more than 5.55, indicating that the fermentation process may not be ideal, resulting in the growth of harmful microorganisms such as mold and bacteria and, subsequently, in feed deterioration and reduced feeding value. However, silage still have a certain value, especially during feed shortages. In view of this situation, it can be speculated that total fermentation can be achieved by adjusting the inoculation amount of lactic acid bacteria; controlling temperature and humidity; or adding molasses, inorganic acid, or other substances to adjust the fermentation process and improve the acidity of silage.

### 4.3. Comprehensive Analysis of Silage Quality Based on Membership Function

In this experiment, according to the membership function analysis results, it can be seen that group B inoculants reduced the NDF and ADF contents of alfalfa to a certain extent and significantly increased the CP and LA contents, which is similar to the conclusions of previous studies on formic acid inoculants and *LP* inoculants [40]. The RFV content in group B was significantly different from that in groups A and D (*p* < 0.05). The RFV is roughly calculated according to the NDF and ADF contents in roughage. The lower the NDF and ADF contents, the better the quality [41]. Therefore, a high RFV in silage is directly related to low a NDF content. Studies [42] have shown that formic acid inoculants can significantly reduce the pH, AN/TN, and ADF of silage (*p* < 0.05). Studies have shown that the addition of *LP* decreased the pH value and the number of *Escherichia coli* in the leaves of four litchi species, while reducing the production of AN (*p* < 0.01) [43]. Formic acid inhibits plant respiration and the fermentation of undesirable microorganisms (such as *Clostridium*, *Bacillus*, etc.), while *Lactobacillus plantarum* inhibits mold and spoilage bacteria by producing lactic acid. It can be concluded that group B silage should also have a similar antibacterial ability to prevent silage deterioration. In addition, formic acid and *Lactobacillus plantarum* can reduce the loss of nutrients in the silage process, such as protein, carotene, vitamin C, etc. [44,45], indicating that group B silage should also be able to effectively preserve nutrients in the feed.

Group B had the best silage quality and the average membership function value was 0.69. Groups A, C, and D exhibited no obvious improvement regarding the nutritional fermentation quality of alfalfa. The authors of [46] conducted an overall evaluation of the whole-plant silage quality of various maize varieties using membership functions. The study pointed out that the membership function values of different varieties were quite close to the relative feeding value after silage, further emphasizing the accuracy of principal component analysis and membership function analysis in the overall evaluation [47]. In addition, some researchers [48] used the method of membership function to evaluate the effect of different inoculants on the quality of corn stalk silage. The results of the study revealed that a ratio of 0.02% cellulase resulted in the best silage quality [49,50,51]. This not only confirmed the significant degradation effect of cellulase on wood fiber components but also indirectly demonstrated the accuracy of this method in the comprehensive evaluation of corn stalk silage [52,53]. Therefore, this method eliminates the bias that may arise when evaluating silage quality from a single index alone [54].

## 5. Conclusions

Different silage agents had different effects on the fermentation quality and nutrient composition of alfalfa silage. The results showed that the addition of exogenous Xinlaiwang I alfalfa silage had significant positive effects on the fermentation quality and nutritional value of alfalfa. In order to give full play to the role of exogenous lactic acid bacteria, improve the overall quality of alfalfa silage, and effectively promote the subsequent production practice, attention should be paid to the screening of *Lactobacillus plantarum* and *Lactobacillus casei* and other strains with strong adaptability and good fermentation effects. In addition, reasonable control of the amount of bactericide is necessary to ensure its effective role in the silage process; it is worth considering its combination with other beneficial inoculants to achieve synergistic effects. This study provides a solid theoretical basis for selecting the best *Lactobacillus* inoculants suitable for alfalfa silage fermentation. However, further experimental studies are needed to validate and refine these conclusions.

## Figures and Tables

**Table 1:** Raw materials nutrition content composition of alfalfa.

DM (%FW)	CP (%DM)	NDF (%DM)	ADF (%DM)	WSC (%DM)	RFV (%)
85.65	25.33	31.84	23.86	5.94	181.33

Note: DM (dry matter): this refers to the residual substance after the removal of water in the feed and is the basis for measuring the nutrient content of the feed. FM (fresh matter): this usually refers to the weight of the plant or feed when it has not been dried, that is, the weight when in a state of high water content. In the feed industry, fresh weight is often used to indicate the weight of fresh feed. Relative feeding value is a measure used to assess the nutritional value of feed, especially for roughage such as hay and silage). CP (crude protein): the general term for nitrogen-containing compounds in feed, including true protein and ammoniates; this is an indicator used to measure the protein content of feed. NDF (neutral detergent fiber): refers to the portion of fiber in feed that cannot be dissolved by neutral detergents, including cellulose, hemicellulose, and lignin and is used to assess the digestibility of feed. ADF (acid detergent fiber): refers to the portion of fiber in the feed that cannot be dissolved by acid detergents, mainly consisting of cellulose and lignin, and is used to further evaluate the digestive properties of the feed. WSCs (water-soluble carbohydrates) refers to water-soluble carbohydrates in feed, including sugars and certain starches, which are a source of rapidly available energy. RFV (relative feed value): the RFV is calculated based on the dry matter digestibility (DDM) and dry matter intake (DMI) of the feed, which reflects the feeding value of the feed relative to standard feed (usually medium-quality forage). The higher the RFV, the higher the nutritional value of the feed, and the more energy and nutrients the animal receives from it.

**Table 2:** Sources, compositions, and contents of silage inoculants.

Microbial Colony/(CFU·g^−1^) and Other Components	Xinlaiwang I Straw Silage Inoculants (A)	Xinlaiwang I alfalfa Silage Inoculants (B)	Zhuanglemei Silage Fermentation Inoculants (C)	Bao Shi Qing (D)	Kangfuqing S Lactic Acid Bacteria Silage Inoculant
*Lactobacillus plantarum*	≥1 × 10^10^	≥1 × 10^11^	≥1.6 × 10^9^	≥2.5 × 10^10^	NC
*Pediococcus pentosaceus*	≥1 × 10^10^	NC	NC	≥2.5 × 10^10^	NC
*Lactobacillus buchneri*	≥1 × 10^10^	NC	≥4.0 × 10^8^	≥2.5 × 10^10^	≥1 × 10^11^
*Lactobacillus casei*	NC	≥1 × 10^11^	NC	NC	NC
Water	NC	NC	<10.0%	NC	NC
Source	Xinlaiwang Nanjing Biotechnology Co., Ltd. (Nanjing, China)	Xinlaiwang Nanjing Biotechnology Co., Ltd.	Sichuan Gaofu Ji Biotechnology Co., Ltd. (Chengdu, China)	Neuman Agricultural Trading Shanghai Co., Ltd. (Shanghai, China)	AiDekang Dalian environmental protection Products Co., Ltd. (Dalian, China)

Note: NC—no content. LAB, lactic acid bacteria; *L. plantarum*, *Lactobacillus plantarum*; *P. pentosaceus*, *Pediococcus pentosaceus*; *L. buchneri*, *Lactobacillus buchneri*; *L. casei*, *Lactobacillus casei*; CFU, colony-forming units.

**Table 3:** Nutrition content composition four alfalfa feed silage types with different LAB treatments.

Items	DM (%FW)	CP (%DM)	NDF (%DM)	ADF (%DM)	WSCs (%DM)	RFV (%)
CK	88.95 b ± 0.06	18.80 c ± 3.65	32.95 ab ± 0.84	26.90 a ± 1.00	1.67 ab ± 0.16	149.33 a ± 4.04
A	89.41 ab ± 0.40	22.93 ab ± 0.35	33.10 ab ± 1.10	26.60 a ± 1.00	1.60 b ± 0.10	144.33 a ± 1.53
B	89.73 b ± 0.06	23.13 ab ± 0.35	30.38 c ± 0.82	24.24 a ± 2.30	1.86 a ± 0.06	165.33 a ± 2.08
C	89.31 ab ± 0.20	20.80 bc ± 1.25	33.47 a ± 0.81	26.83 a ± 0.84	1.65 ab ± 0.05	152 a ± 23.90
D	89.45 ab ± 0.28	23.83 ab ± 0.35	31.59 bc ± 0.46	25.17 a ± 1.04	1.56 b ± 0.21	143 a ± 27.40
E	89.59 ab ± 0.40	24.70 a ± 0.95	32.52 ab ± 0.50	26.67 a ± 1.60	1.74 ab ± 0.13	136 a ± 22.34

Note: Different lowercase letters in the same column indicate a significant difference (*p* < 0.05). The same lowercase letter means no significant difference (*p* > 0.05).

**Table 4:** Effects of different lactic acid bacteria treatments on the quality of alfalfa silage (% DM).

Items	pH	AN/TN (%)	LA (g/kg DM)	AA (g/kg DM)	PA (g/kg DM)	BA (g/kg DM)
CK	6.48 a ± 0.13	10.59 ab ± 1.83	0.46 c ± 0.79	34.98 b ± 5.62	ND	ND
A	6.44 a ± 0.23	11.62 ab ± 0.64	2.88 c ± 2.64	37.09 b ± 5.00	ND	ND
B	5.57 b ± 0.03	10.35 b ± 1.84	14.82 b ± 0.61	54.53 a ± 7.95	ND	ND
C	6.70 a ± 0.34	11.30 ab ± 2.06	1.77 c ± 3.06	56.53 a ± 3.87	ND	ND
D	5.82 b ± 0.35	13.40 a ± 1.00	26.84 a ± 13.36	22.25 b ± 19.31	ND	ND
E	5.55 b ± 0.03	10.41 b ± 1.45	13.87 b ± 1.01	30.46 b ± 4.26	ND	ND

Note: ND means not detected (Possibility 1: Low actual levels. We will explore the possibility that the actual levels of PA and butyric acid in the sample are so low as to be below the sensitivity threshold of the detection method. This may be due to factors such as the characteristics of feed materials, fermentation conditions during silage, or storage conditions. Possibility 2: Absence. We will also consider the possibility that these acids are not present in the sample at all. This may be due to specific silage processes or additives used that inhibit the production of these acids. The possibility of using more sensitive detection methods, such as GC-MS, should be considered in future studies). LA, lactic acid; AA, acetic acid; PA, propionic acid; BA, butyric acid; AN/TN, ammonia nitrogen/total nitrogen. Different lowercase letters in the same column indicate significant differences (*p* < 0.05). The same lowercase letters mean no significant differences (*p* > 0.05).

**Table 5:** Effects of different lactic acid bacteria treatments on the nutrition and fermentation quality of alfalfa silage.

Items	DM (%FW)	CP (%DM)	LA (g/kg DM)	WSCs (%DM)	RFV (%)	AA (g/kg DM)	NDF (%DM)	ADF (%DM)	pH	AN/TN (%)	MEAN	RANK
CK	0	0	0.54	0.37	0.45	0.63	0.17	0	0.98	1	0.41	4
A	0.59	0.7	0.09	0.13	0.28	0.57	0.12	0.11	0.23	0.58	0.34	5
B	1	0.73	0	1	1	0.06	1	1	0.19	0.92	0.69	1
C	0.46	0.34	0.05	0.3	0.55	0	0	0.03	0	0.69	0.24	6
D	0.64	0.85	1	0	0.24	1	0.61	0.65	0.77	0	0.58	3
E	0.82	1	0.51	0.6	0	0.76	0.31	0.09	1	0.98	0.61	2

## Data Availability

The original contributions presented in the study are included in the article, further inquiries can be directed to the corresponding authors.

## Abbreviations

The following abbreviations are used in this manuscript:

FM	Fresh matter
DM	Dry matter
CP	Crude protein
RFV	Relative feeding value
NDF	Neutral detergent fiber
ADF	Acid detergent fiber
WSC	Water soluble carbohydrate
ASH	Crude ash
LA	Lactic acid
AA	Acetic acid
PA	Propionic acid
BA	Butyric acid
LAB	Lactic acid bacteria
*L. plantarum*	*Lactobacillus plantarum*
*P. pentosaceus*	*Pediococcus pentosaceus*
*L. buchneri*	*Lactobacillus buchneri*
*L. casei*	*Lactobacillus casei*
CFU	Colony-forming units
